# Larger genomes show improved buffering of adult fitness against environmental stress in seed beetles

**DOI:** 10.1098/rsbl.2022.0450

**Published:** 2023-01-25

**Authors:** Jesper Boman, Göran Arnqvist

**Affiliations:** ^1^ Evolutionary Biology, Department of Ecology and Genetics, Uppsala University, Uppsala, Sweden; ^2^ Animal Ecology, Department of Ecology and Genetics, Uppsala University, Uppsala, Sweden

**Keywords:** genome size, transposable elements, canalization, thermal adaptation, gene duplication, Bruchinae

## Abstract

Our general understanding of the evolution of genome size (GS) is incomplete, and it has long been clear that GS does not reflect organismal complexity. Here, we assess the hypothesis that larger genomes may allow organisms to better cope with environmental variation. It is, for example, possible that genome expansion due to proliferation of transposable elements or gene duplications may affect the ability to regulate and fine-tune transcriptional profiles. We used 18 populations of the seed beetle *Callosobruchus maculatus*, which differ in GS by up to 4.5%, and exposed adults and juveniles to environmental stress in a series of experiments where stage-specific fitness was assayed. We found that populations with larger genomes were indeed better buffered against environmental stress for adult, but not for juvenile, fitness. The genetic correlation across populations between GS and canalization of adult fitness is consistent with a role for natural selection in the evolution of GS.

## 1. Introduction

The causal factors underlying the evolutionary dynamics of variation in genome size (GS) remain enigmatic. A long-standing belief is that the efficacy of natural selection to counter slightly deleterious GS expansion may vary across lineages [1,2], but proxies of the efficacy of natural selection are not generally and obviously related to GS ([3,4], but see [5]). The fact that GS correlates with important organismal properties, such as metabolic rate [6], reproductive fitness [7], survival and life-history traits [8], developmental timing [9] and organismal growth [10], suggests that natural selection may play a more direct role in GS evolution. Natural selection could act on GS variation in several non-mutually exclusive ways [1,2].

Here, we entertain the possibility that larger genomes may sometimes be favoured by selection because they allow organisms to better cope with environmental variation. This would essentially result in fitness being more canalized, and this could in part be due to gene duplications, allowing functional buffering against deleterious mutations [11], but perhaps primarily due to the gene regulatory machinery being more fine-tuned in lineages with larger genomes [12]. GS expansion is closely related to transposable element (TE) proliferation in many groups [2,5,13,14], and several types of TEs are well known to affect plasticity in gene expression in a variety of ways [15,16], suggesting a possible route by which such effects could occur [7]. In essence, larger genomes could allow organisms to respond to environmental conditions and adaptively regulate underlying physiological processes and metabolic pathways, through differential transcription, post-transcriptional modification and/or translation [15,16], resulting in life-history traits and fitness being more canalized. Direct empirical assessments of this hypothesis are few but encouraging. First, in a series of controlled laboratory experiments, Ellis *et al*. [8] found that GS was indeed related to phenotypic plasticity in thermal sensitivity in several life-history traits across distinct *Drosophila melanogaster* genotypes. Second, in comparative studies, species with larger genomes have been found to have larger environmental and geographical distributions in bacteria [17], birds [18] and caddisflies [19], consistent with larger genomes being better able to produce viable phenotypes under a wider range of environments.

Studies of intraspecific variation in GS suffer less from confounding effects associated with large phylogenetic distances [2] and we thus employ a population-level approach here: we ask whether populations of the seed beetle *Callosobruchus maculatus* with larger genomes are better buffered against environmental stress. A previous study of this species [7] documented sizeable variation in GS across populations and showed that variation in GS was related to certain components of environment-specific reproductive fitness, namely female fecundity and male competitive fertilization success. Here, we expose these populations to environmental stress and assay key fitness components in both adults and juveniles.

## 2. Methods

We used 18 distinct laboratory populations of the granivorous seed beetle *C. maculatus* (Coleoptera, Bruchinae) that were originally collected at different geographical locations in Asia, Africa and North and South America. These populations show sizeable genetic differentiation (*F*_ST_ = 0.23–0.26) but are reproductively compatible in the sense that they produce viable offspring, although egg-to-adult survival is typically somewhat lower in between-population crosses (approx. 80% versus approximately 90% within populations) [20]. Populations were collected at various points in time (1975–2010), but the year of collection was not related to GS (*r* = −0.13, *p* = 613) and accounting for year did not significantly affect any of the buffering effects discussed below (*p* > 0.1 in all cases). Average GS in these populations was determined by Arnqvist *et al*. [7], using flow cytometry, who showed that GS differs highly significantly and by some 4.5% across populations (male GS range: 1.17–1.23 Gbp; [7]). Because GS does not show any phylogenetic signal across these populations [7], we did not control for phylogenetic independence here. For more information on these laboratory populations, GS estimation and rearing conditions, we refer to Arnqvist *et al*. [7].

We conducted a series of standardized fitness assays, replicated independently in each of the 18 populations. These are briefly described below. See electronic supplementary material methods for a more detailed account of the methods.

### (a) Effects of food stress on adult fitness

Virgin adult males and females were placed in pairs in Petri dishes provided with a superabundant supply of *Vigna unguiculata* beans. Adults were provided either with (i) food (pollen) and sugar water, (ii) only water or (iii) no food or water (i.e. aphagy) throughout their typically 5–15 day adult life, and we subsequently recorded the lifetime offspring production (number of adult offspring produced) of each pair (*N*_total_ = 465).

### (b) Effects of food stress on juvenile fitness

Recently emerged adult males and females (*N* = 15 of each sex) were placed in oviposition jars supplied with 80 beans of either (i) *V. unguiculata*, (ii) *V. angularis* or (iii) *Cicer arietinum* at 29°C and allowed to ovipisit for 4 h. These host beans contain different types and amounts of plant defense compounds that provide varying degrees of digestive challenges to *C. maculatus* larvae. The preferred larval host is *V. unguiculata,* while especially *C. arietinum* is challenging as a food resource for larvae. Following oviposition, the adults were removed and beans with eggs were placed individually under 29°C. Hatching was monitored by frequent spot checks (1–2 times per day) and we determined sex, body weight and development time for all hatching offspring (*N*_total_ = 2255).

### (c) Effects of thermal stress on juvenile fitness

Recently emerged adult males and females (*N* = 30 of each sex) were placed in an oviposition jar supplied with 200 *V. unguiculata* at 29°C for 4 h, as in the experiment above. The adults were then removed and beans with eggs were placed individually under either (i) 22°C, (ii) 29°C or (iii) 35°C. Hatching was again monitored by frequent spot checks and we determined sex, body weight and development time for all hatching offspring (*N*_total_ = 2663).

### (d) Analysis

We first tested for difference between populations in buffering of fitness in general linear models including data for all populations. The strength of the effect of environmental stress on fitness in each population (i.e. the effect size) was then determined as the *F*-ratio of the environmental treatment effect in population-specific linear models of fitness, which we refer to here as fitness buffering. Note that a high value corresponds to low buffering. The models of adult fitness included only environmental treatment, while treatment, sex and their interactions were included in models of juvenile body weight and development time. The treatment effect on juvenile growth rate was determined in models of body weight, including treatment, sex and their interactions as factors and development time as a covariate. All observations with an absolute value of the standardized residual |R| > 3 were deemed outliers and excluded from analyses. Population-specific mean fitness was determined as the marginal mean of each fitness component.

## 3. Results

**Figure 1.**
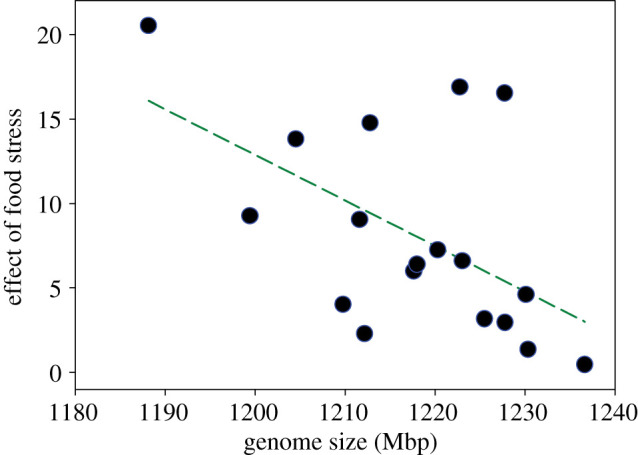
GS is negatively correlated with the effect size (*F*-value) of food stress on adult lifetime offspring production across populations, such that populations with larger genomes are better able to buffer their fitness across environmental conditions (bootstrap [9999 replicates] mean and bias corrected 95% CI for *r*: −0.89 — −0.52 — −0.06; permutation test of *H*_0_: *r* = 0: *p* = 0.017). Dashed line represents LS regression line (*p* = 0.018; table 1).

Populations varied markedly in the effects of food stress on adult fitness (treatment × population interaction; *F*_34,408_ = 2.95, *p* < 0.001) and in the effects of both nutritional and thermal stress on all juvenile fitness components measured (treatment × population interaction; *p* < 0.001 in all cases; electronic supplementary material, table S1). The analyses of covariation between GS and the amount of buffering of fitness across populations are presented in table 1. We found that adult fitness is less affected by food stress in populations with relatively large genomes (figure 1), in line with our prediction. By contrast, none of our six measures of buffering of juvenile fitness was significantly related to GS (table 1), despite sizeable differences between populations in the effects of environmental stress on juvenile fitness components (electronic supplementary material, table S1). Moreover, adult fitness buffering was not significantly related to any measure of juvenile fitness buffering (2 stressors × 3 components; all |*r*| < 0.39; all *p* > 0.110).

**Table 1.** Linear models using GS to predict the strength of the effect of environmental stress on fitness components across populations (*N* = 18).

fitness component	*β*	s.e.*_β_*	*t*	*p*
adult lifetime fecundity (food)	−0.27	0.10	2.63	0.018
juvenile body weight (temperature)	1.64	1.00	1.63	0.122
juvenile body weight (food)	0.25	0.56	0.45	0.660
juvenile development time (temperature)	−10.76	140.58	0.07	0.939
juvenile development time (food)	2.37	1.66	1.43	0.172
juvenile growth rate (temperature)	0.19	0.26	0.72	0.479
juvenile growth rate (food)	0.07	0.53	0.15	0.883

We found no support for a general relationship between GS and overall mean fitness across all environments here. GS was not significantly related to juvenile fitness: none of the population-specific marginal means of juvenile fitness components was significantly correlated with GS (all six |*r*| < 0.15; all *p* > 0.562). Average adult fitness (marginal mean offspring production over treatment levels) was also not significantly correlated with GS (*r* = −0.29; *p* = 0.231) or with buffering of adult fitness (*r* = 0.20; *p* = 0.422). Arnqvist *et al*. [7] documented a positive association between GS and certain measures of reproductive fitness in the populations studied here but found the relationship between GS and lifetime offspring production under aphagous conditions to be positive but non-significant, which was true also in the current experiments (*r* = 0.06; *p* = 0.805). However, variation in adult mean fitness across populations was environment-dependent to a large extent: although fitness in the water-only treatment was significantly correlated with fitness in the aphagy treatment (r = 0.60; *p* = 0.008), none were correlated with fitness in the food-and-water treatment (*r* = 0.22; *p* = 0.359 and *r* = −0.09; *p* = 0.732, respectively).

## 4. Discussion

Needless to say, our findings do not refute the possibility that several different factors and mechanisms, many of which are often classified as non-adaptive [1,2], have contributed to the divergent evolution of GS seen in the populations studied here. They do, however, support the tenet that natural selection has contributed to the evolution of GS. In particular, our results are consistent with the hypothesis that larger genomes allow improved buffering of adult fitness against environmental stress. Although the proximate reasons for this pattern are not addressed here, we suggest that the flexibility of the transcriptional or post-transcriptional machinery may contribute. The repeat content of the *C. maculatus* genome is as high as 71% [21] and annotation of the repetitive elements has shown that they belong to a variety of DNA transposons, LINEs, SINEs, LTR retrotransposons and satellite DNA [20]. The most abundant superfamily is Tc1/Mariner, within the class of DNA transposons, which makes up some 10% of the *C. maculatus* genome. TEs in this superfamily, as well as those in several other classes, are known to affect gene regulation in a variety of different ways [15,22,23]. Differences between seed beetle lineages in GS seem to be due primarily to variation in the degree of expansion of repeat elements [7,21]. In addition, gene duplications could contribute to the relationship seen between GS and adult fitness buffering. In a study on the green peach aphid, *Myzus persicae*, Mathers *et al*. [24] used RNA interference-mediated knock-down to show that a family of duplicated genes was involved in the degree of host generalism. In this species, buffering of fitness across different host plants was apparently conferred by gene duplications. It is thus possible that a richer palette of non-coding DNA and/or gene family expansions in populations with larger genomes allows a more responsive physiological machinery that results in improved buffering under environmental stress. Future studies of differential gene expression or protein abundance under environmental stress in these populations would allow an assessment of these possibilities.

While we found significantly improved buffering of adult lifetime offspring production, this was not true for juvenile fitness components. Although juvenile development time and adult body size are both related to fitness in *C. maculatus*, life table analyses show that adult lifetime offspring production is most intimately linked to net fitness (e.g. [25]). Because natural selection should act to render fitness components that contribute most to net fitness to be better buffered against environmental perturbations [26], this may contribute to our findings. To the extent that GS reflects adaptations that allow canalization of fitness, selection for adult fitness buffering may simply have been stronger than selection for buffering juvenile performance.

## Data Availability

All data have been archived and are available at Mendeley Data: https://doi.org/10.17632/4kmtdh8dmn.1 [27]. Supplementary material is available online [28].
